# Venous Thromboembolism Prophylaxis in Major Orthopedic Surgeries and Factor XIa Inhibitors

**DOI:** 10.3390/medsci11030049

**Published:** 2023-08-11

**Authors:** Aaryana Jones, Rami A. Al-Horani

**Affiliations:** Division of Basic Pharmaceutical Sciences, College of Pharmacy, Xavier University of Louisiana, New Orleans, LA 70125, USA; ajone131@xula.edu

**Keywords:** orthopedic, thromboprophylaxis, VTE, LMWHs, oral anticoagulants, aspirin, factor XIa, milvexian, osocimab, abelacimab

## Abstract

Venous thromboembolism (VTE), comprising pulmonary embolism (PE) and deep vein thrombosis (DVT), poses a significant risk during and after hospitalization, particularly for surgical patients. Among various patient groups, those undergoing major orthopedic surgeries are considered to have a higher susceptibility to PE and DVT. Major lower-extremity orthopedic procedures carry a higher risk of symptomatic VTE compared to most other surgeries, with an estimated incidence of ~4%. The greatest risk period occurs within the first 7–14 days following surgery. Major bleeding is also more prevalent in these surgeries compared to others, with rates estimated between 2% and 4%. For patients undergoing major lower-extremity orthopedic surgery who have a low bleeding risk, it is recommended to use pharmacological thromboprophylaxis with or without mechanical devices. The choice of the initial agent depends on the specific surgery and patient comorbidities. First-line options include low-molecular-weight heparins (LMWHs), direct oral anticoagulants, and aspirin. Second-line options consist of unfractionated heparin (UFH), fondaparinux, and warfarin. For most patients undergoing knee or hip arthroplasty, the initial agents recommended for the early perioperative period are LMWHs (enoxaparin or dalteparin) or direct oral anticoagulants (rivaroxaban or apixaban). In the case of hip fracture surgery, LMWH is recommended as the preferred agent for the entire duration of prophylaxis. However, emerging factor XI(a) inhibitors, as revealed by a recent meta-analysis, have shown a substantial decrease in the occurrence of VTE and bleeding events among patients undergoing major orthopedic surgery. This discovery poses a challenge to the existing paradigm of anticoagulant therapy in this specific patient population and indicates that factor XI(a) inhibitors hold great promise as a potential strategy to be taken into serious consideration.

## 1. Introduction

Venous thromboembolism (VTE), encompassing pulmonary embolism (PE) and deep vein thrombosis (DVT), is a serious complication during and after a hospital stay, particularly in surgical patients who undergo general anesthesia. In fact, it appears that >50% of hospitalized patients are at risk for VTE [1]. The risk of VTE remains elevated for up to 2 months after noncancer general surgery [2]. Among all patients, orthopedic patients undergoing major surgeries appear to be at a higher risk for PE and DVT. These major orthopedic surgeries comprise total or partial knee replacement surgery, total or partial hip replacement surgery, and hip fracture surgery involving open reduction and internal fixation [3,4,5,6,7]. For instance, studies have indicated that the incidence of DVT among hospitalized patients ranges from 10% to 40%. Nevertheless, the incidence of DVT escalates to 40–60% in patients undergoing major orthopedic surgery [8]. Likewise, in the absence of preventive measures, PE accounts for 5–10% of deaths among hospitalized patients. Estimates suggest that the occurrence of fatal PE in hospitalized patients ranges from 0.1% to 0.8% following elective general surgery, but it can increase to 3.0% after elective hip replacement and up to 7.0% following hip fracture surgery [9,10,11,12]. In some cases of foot and ankle surgery, DVT may develop without clinically apparent symptoms or signs [13].

Another set of recent studies indicated that, without pharmacologic thromboprophylaxis, the rate of DVT detected with routine contrast venography was about 54% after total hip arthroplasty, of which 27% were proximal DVT. This rate was about 64% after total knee arthroplasty, of which 15% were proximal DVT [14]. Symptomatic VTE, which is of greater clinical significance, was observed at a range of 2.0% to 3.0% following total hip arthroplasty without pharmacologic prophylaxis. With pharmacologic prophylaxis, the occurrence declined to approximately 1.0% to 1.2%, with specific breakdowns for total hip arthroplasty showing PE at 0.3% and DVT at 0.7%, and for total knee arthroplasty showing PE at 0.3% and DVT at 0.9% [3,15,16,17].

Although VTE can develop after any major surgical procedure, orthopedic patients are particularly more vulnerable due to the presence of all the pathophysiologic processes outlined in Virchow’s triad. These processes include (1) the use of a tourniquet, bed rest, and immobilization, which contribute to venous blood stasis; (2) trauma, which leads to an increase in thromboplastin agents; (3) surgical limb manipulation, resulting in endothelial vascular injuries; and (4) the utilization of polymethylmethacrylate bone cement, which increases hypercoagulability [18]. Thus, VTE prophylaxis is of the utmost importance for patients undergoing orthopedic surgery or those with orthopedic trauma.

Overall, major orthopedic surgery involving the lower extremities carries a heightened risk of symptomatic VTE compared to many other surgical procedures, with an estimated incidence of about 4%. This risk is most pronounced during the initial 7 to 14 days following surgery. Furthermore, major orthopedic surgeries also exhibit a higher likelihood of significant bleeding complications in comparison to other surgeries, with an estimated rate of 2–4%. The presence of additional individual risk factors, such as a history of previous VTE or conditions such as thrombocytopenia, can further amplify the risks of both VTE and bleeding, respectively [3,5,6,7,19,20,21,22]. Therefore, several thrombosis prophylactic methods have been evaluated in orthopedic patients. These include pharmacological methods and mechanical methods.

## 2. Current Methods

### 2.1. Pharmacological Methods

A variety of antithrombotics are available for the prevention, management, and treatment of thromboembolism in orthopedic surgeries. These include agents that prevent platelet activation, aggregation, and/or adhesion, known as antiplatelets, and others that inhibit clotting factors, known as anticoagulants. When choosing a pharmacological thromboprophylaxis agent for patients undergoing major lower-extremity orthopedic procedures, who possess a low risk of bleeding, factors such as the specific type of surgery and comorbidities play a role in the decision-making process [23,24,25,26,27].

Consistent with the American College of Chest Physicians and the American Society of Hematology Recommendations, patients undergoing major lower-extremity orthopedic surgery, who have a low risk of bleeding, are recommended to receive pharmacological thromboprophylaxis. Additionally, the use of intermittent pneumatic compression (IPC) devices may be considered as an adjunct to pharmacological prophylaxis [3,4,5,6,7]. In cases where patients have contraindications to pharmacological thromboprophylaxis, it is recommended that they solely receive mechanical prophylaxis until the contraindication is resolved. First-line agents for patients undergoing total hip or total knee arthroplasty include LMWHs (enoxaparin, dalteparin, tinzaparin, nadroparin, danaparoid, or ardeparin) or a direct oral anticoagulant as the initial agent in the early perioperative period (up to 14 days). Among the direct oral anticoagulants, the use of rivaroxaban or apixaban, rather than edoxaban or dabigatran, is prioritized for thromboprophylaxis in this specific setting. The reason for this recommendation is that there is a greater amount of data available to support the use of rivaroxaban or apixaban in this context. It is also important to note that aspirin is not recommended as the sole initial agent for thromboprophylaxis during the early postoperative period. First-line agents for patients undergoing hip fracture surgery are LMWHs for the entire duration of prophylaxis (up to 35 days). In patients with severe renal failure, unfractionated heparin (UFH) appears to be the agent of choice. In cases when UFH injections are undesirable, warfarin may be considered as an alternative option. Nevertheless, it is important to note that patients with heparin-induced thrombocytopenia (HIT) or a history of HIT should be treated with a non-heparin agent [3,4,5,6,7,23,24,25,26,27].

The optimal timing for administering pharmacological thromboprophylaxis in patients undergoing major orthopedic surgery remains uncertain, as it is influenced by various factors, including the specific agents chosen for prophylaxis. In this context, when utilizing LMWHs for thromboprophylaxis, the recommended approach is to administer an initial dose at least 12 h before surgery and then to continue with a second dose at least 12 h after surgery. Alternatively, LMWHs can be started postoperatively with the first dose ≥12 h after surgery. The same approach is exploited when using UFH. As a rule of thumb, LMWHs are not to be administered close to surgery (within four hours preoperatively and within four hours postoperatively) to avoid elevating the bleeding risk [28,29]. Fondaparinux is to be started six or more hours after skin closure. Nevertheless, fondaparinux’s first dose is recommended 8–12 h postoperatively to mitigate the bleeding risk [3,4,5,6,7]. When it comes to oral agents such as direct oral anticoagulants and warfarin, they are typically initiated postoperatively, usually 6 to 12 h or even longer after surgery, provided that the patients are able to consume food.

Considering the duration, it is recommended to continue thromboprophylaxis with an anticoagulant agent for a minimum duration of 10–14 days. Nevertheless, the duration can be adjusted to be longer or shorter depending on factors such as the specific surgery and other individual risk factors [30,31,32,33,34,35,36,37,38,39,40,41,42,43,44,45,46,47]. For instance, in patients undergoing hip arthroplasty, a total duration of 35 days of thromboprophylaxis is recommended. Typically, an LMWH or direct oral anticoagulant is utilized throughout the entire 35-day course of thromboprophylaxis. However, for specific lower-risk patients, a transition to aspirin is now being recommended after 5–10 days of initial therapy, which is then continued for the remaining duration of the 35-day course. In the case of patients undergoing knee arthroplasty, it is generally not advised to continue thromboprophylaxis beyond 14 days, as long as the patient is capable of walking independently by the end of this period. For lower-risk patients, transitioning to aspirin alone after five days of initial therapy is being suggested for the remaining 10–14-day course. For patients who do not fall into the aforementioned lower-risk category, it is advised to maintain thromboprophylaxis with an LMWH or direct oral anticoagulant throughout the entire 10–14-day period. In cases where patients are not able to walk independently by the end of 14 days, the use of an LMWH or direct oral anticoagulant prophylaxis should continue until they achieve full ambulation or for a total of 35 days. In the context of patients undergoing hip fracture surgery, thromboprophylaxis with the same agent (LMWH or direct oral anticoagulant) is recommended for the entire duration of 35 days, although aspirin may sometimes be considered as an alternative [30,31,32,33,34,35,36,37,38,39,40,41,42,43,44,45,46,47].

#### Specific Agents

**UFH**

UFH is considered a second-line agent for thromboprophylaxis in orthopedic surgery. UFH has been used as an anticoagulant in various forms since its discovery by McLean in 1916 [48]. Mechanistically, UFH activates a natural plasma-based anticoagulant, antithrombin, and makes a faster inhibitor of some clotting factors such as thrombin, factor Xa, factor IXa, and to some extent factor XIIa. By inhibiting thrombin, UFH prevents fibrin formation and inhibits the thrombin-induced activation of factor V and factor VIII [48]. 

Chemically, UFH is a heterogeneous and polydisperse mixture of sulfated glycosaminoglycans, with an average molecular weight of approximately 15 kDa. Only one-third of UFH has the pentasaccharide DEFGH that is responsible for its anticoagulant activity by recognizing the corresponding binding site on antithrombin. Certain sulfate groups on this pentasaccharide are the key functional groups contributing to its pharmacological action. UFH is parenterally used (IV or SC), has a rapid onset of action, and its half-life is relatively short [49,50,51].

The measurement of therapeutic levels of heparin is determined by the activated partial thromboplastin time (APTT). Achieving therapeutic levels (APTT of 1.2–1.5 times the control value) is more likely with continuous IV infusion due to the rapid clearance of heparin from the systemic circulation. UFH can serve as an alternative option that can be used as a second-line choice for patients who are unable to receive LMWHs or direct oral anticoagulants, such as those with renal failure. UFH is given at a dose of 5000 units SC twice daily or three times daily [52]. This low-dose UFH regimen results in a 60–70% reduction of DVT and PE in low- or moderate-risk patients. Nevertheless, this method is not as effective in patients who are at high risk for the development of DVT or PE. Studies have demonstrated a high hemorrhagic complication rate of 8–15% when UFH is used for postoperative DVT prophylaxis [53,54]. In patients with obesity, the optimal dose is unknown, although 7500 units twice daily can be used [55,56]. No renal adjustment is necessary.

In particular, the effectiveness of UFH in orthopedic settings has been extensively studied through randomized trials conducted from the 1970s to the 1990s. A meta-analysis of eight trials, which included more than 500 patients undergoing hip arthroplasty, demonstrated that UFH reduced the occurrence of DVT by 44% compared to no thromboprophylaxis (RR 0.53, 95% CI 0.32–0.89). Likewise, consistent results were observed in trials involving patients undergoing hip fracture surgery (RR 0.56, 95% CI 0.39–0.81) with UFH administration, as reported in six trials [3,22]. 

Heparin overaction is counteracted with protamine sulfate. Approximately 1 milligram of protamine sulfate can neutralize around 100 units of UFH activity [57]. Protamine must be administered very slowly by IV infusion over a 10 min period, ensuring that doses do not exceed 50 mg. The required amount of protamine decreases rapidly as the time from the initial heparin administration increases. The final dosage is determined based on clotting time measurement [58]. UFH has certain limitations, including nonspecific binding that leads to variable pharmacokinetics, the need for monitoring and adjusting the dosage based on the APTT, a short half-life, and the absence of an oral formulation. Furthermore, 2–4% of patients may develop HIT, an antibody-mediated adverse reaction that can result in venous and arterial thrombosis. Severe cases of HIT can lead to disseminated intravascular coagulation and gangrene. Danaparoid sodium or recombinant hirudin can be effective treatments in life-threatening cases [59]. A comparison study comparing the outcomes of thromboprophylaxis between enoxaparin (LMWH) and UFH revealed a 74% lower incidence of VTE in the enoxaparin group, with no significant difference in side effects, in-hospital deaths, or economic factors [60].

**Fondaparinux**

Fondaparinux, a synthetic sulfated pentasaccharide of UFH acting as an indirect factor Xa inhibitor, is a second-line agent for VTE prophylaxis in patients undergoing major lower-extremity orthopedic surgery. It is an option for patients with HIT. Fondaparinux is given at a dose of 2.5 mg SC once daily. Fondaparinux is contraindicated in patients who weigh <50 kg and is avoided in patients with renal insufficiency [57].

The effectiveness of fondaparinux has been extensively studied in various randomized trials and meta-analyses [3,58,59,60,61,62,63,64,65,66,67]. In a meta-analysis conducted in 2016, which included 25 trials with a total of 21,000 patients, fondaparinux demonstrated a significant reduction in the incidence of symptomatic VTE compared to a placebo (0.2% vs. 1.2%; RR 0.15, 95% CI 0.06–0.36). However, it was associated with an increased risk of major bleeding (1.2% vs. 0.5%; RR 2.56; 95% CI 1.48–4.44) [64]. In trials comparing fondaparinux with LMWHs, the rates of symptomatic VTE were similar between the two groups (0.6% in each; RR 1.03; 95% CI 0.65–1.63). However, the incidence of major bleeding events was higher with fondaparinux (2.5% vs. 1.8%; RR 1.38; 95% CI 1.09–1.75) [64]. Fondaparinux has also been compared with direct oral anticoagulants. Retrospective studies reported that direct oral anticoagulants (such as rivaroxaban and/or edoxaban) resulted in lower rates of VTE and either a similar or improved safety profile compared to fondaparinux [68,69]. The use of fondaparinux for extended duration thromboprophylaxis was investigated in a randomized trial involving 656 patients undergoing hip fracture surgery. The study found that compared to a 1-week treatment course, a 1-month course of fondaparinux significantly reduced the incidence of symptomatic VTE (0.3% vs. 2.7%). However, there was a nonsignificant trend toward an increase in major bleeding with extended therapy [70].

**Vitamin K Antagonists: Warfarin**

Warfarin is considered a secondary option for prophylaxis against VTE following major orthopedic surgery. Its use is typically reserved for patients who cannot take other direct oral anticoagulants or LMWHs, such as those with severe renal insufficiency. The mechanism of action of warfarin involves blocking the function of the vitamin K epoxide reductase complex in the liver. This leads to a depletion of the reduced form of vitamin K, which serves as a cofactor for the gamma carboxylation of vitamin-K-dependent coagulation factors. Without gamma carboxylation, the vitamin-K-dependent factors, including factors II, VII, IX, and X, are unable to bind the calcium and phospholipid membranes necessary for their hemostatic function. Warfarin also inhibits the gamma carboxylation of the anticoagulant protein S and protein C, which normally inhibit activated factors VIII and V [71,72].

Structurally, warfarin is a coumarin derivative and exists as a racemic mixture of R and S forms. It is rapidly absorbed from the gastrointestinal tract and extensively bound to plasma proteins. Although warfarin has high bioavailability, it requires 36–72 h to reach a stable loading dose. The dose response to warfarin can vary among patients and is influenced by environmental and genetic factors. Furthermore, its pharmacokinetics can be affected by numerous drug interactions and disease states. Due to these considerations, continuous laboratory monitoring is necessary for warfarin therapy. The effectiveness of warfarin anticoagulation is assessed by measuring the prothrombin time (PT) against a standard control. The international normalized ratio (INR) has replaced the PT for hospital use as it employs a standardized PT, enabling comparisons between different hospitals and laboratories [71,72].

The typical dosing regimen for warfarin involves an initial oral dose of 5 mg once daily. Warfarin is initiated 12 to 24 h after surgery or the evening before the surgery. While some experts adjust the dosage based on a target INR value of 1.5 to 2.5, most experts aim for a target INR of 2.5 (with a range of 2 to 3) [73,74,75]. In patients undergoing major hip surgery, particularly hip fracture surgery, anticoagulation with warfarin has demonstrated a 55% reduction in the incidence of asymptomatic VTE compared to no thromboprophylaxis [22,76,77]. Notably, in one trial, warfarin was found to be more effective than aspirin in preventing DVT following hip fracture surgery [76]. Furthermore, in the majority of trials, warfarin has shown greater efficacy than mechanical methods of thromboprophylaxis [78,79,80,81]. However, more recent clinical trials and meta-analyses have reported the inferiority of warfarin compared to LMWHs.

Warfarin use is associated with several disadvantages. These include a delayed onset of action, requiring time to achieve the therapeutic effect. Additionally, frequent monitoring of INR values is necessary to ensure a consistent and appropriate response to treatment. Warfarin has a long half-life, which may require the administration of vitamin K in cases of bleeding incidents. Another challenge with warfarin is its potential for interactions with other medications and food, which can affect its effectiveness. Moreover, patient response to warfarin can vary widely, making individual dosing adjustments necessary. Hemorrhagic complications have been reported in approximately 3–5% of patients receiving warfarin prophylaxis [71,72].

In contrast to UFH, fondaparinux, and warfarin, the first-line options for anticoagulant VTE prophylaxis after partial or total hip arthroplasty and partial or total knee arthroplasty are LMWHs, selected direct oral anticoagulants (rivaroxaban or apixaban), and aspirin started after an initial 5-day treatment with rivaroxaban or LMWHs. Likewise, the first-line agents for anticoagulant VTE prophylaxis after hip fracture surgery are LMWHs.

**LMWHs**

Multiple randomized trials have established the effectiveness of LMWHs as first-line agents for VTE prophylaxis in patients undergoing major orthopedic surgery on the lower extremities. These studies have consistently demonstrated lower rates of symptomatic VTE when compared to a prophylactic dose of UFH or therapeutic anticoagulation with warfarin. Importantly, the use of LMWHs has shown comparable bleeding rates to these other treatment options.

LMWHs are derived from UFH through chemical or enzymatic fractionation processes, leading to shorter glycosaminoglycan sequences with fewer sulfate groups. These modifications result in an average molecular weight of 4.5 kDa for LMWHs. LMWHs have a greater tendency to activate antithrombin specifically to inhibit factor Xa, whereas UFH activates antithrombin to inhibit both factor Xa and thrombin. Additionally, LMWHs exhibit a longer half-life of approximately 4 h, compared to the shorter half-life of 1 h observed with UFH. Similar to UFH, LMWH overaction can be, although partially, counteracted with protamine sulfate.

In preclinical investigations, LMWHs demonstrated a lower incidence of microvascular bleeding compared to UFH. However, the findings from these studies have not been consistently replicated in human trials. When compared to a placebo, LMWHs have shown a substantial risk reduction of 70–80% for DVT in high-risk orthopedic patients, without an associated increase in major bleeding. Meta-analyses that have examined various methods of DVT prophylaxis, including low-dose UFH, adjusted-dose heparin, and warfarin, have also reported improved DVT prevention with LMWHs, without an elevated risk of bleeding complications [82,83,84]. Importantly, LMWHs are typically avoided in patients with severe renal failure. Dose adjustment is also necessary for patients who have obesity. Several LMWHs are commercially available (Table 1) [85,86,87,88,89,90,91].

Numerous clinical trials have been conducted to assess the effectiveness of LMWHs in comparison to various agents such as placebos, direct oral anticoagulants, UFH, and warfarin. In randomized placebo-controlled trials conducted between the 1980s and 1990s, involving patients undergoing total hip arthroplasty, hip fracture surgery, and total knee arthroplasty, LMWHs consistently demonstrated a reduction in asymptomatic VTE incidence by approximately 50% [92,93,94,95,96]. Furthermore, most trials have indicated that LMWHs lower the risk of VTE by approximately 33% compared to warfarin [97,98,99,100,101,102,103,104,105,106,107]. While earlier studies reported a higher incidence of major bleeding events associated with LMWHs, subsequent studies examining extended thromboprophylaxis did not find significant differences in bleeding risk [83]. Furthermore, in patients undergoing major orthopedic surgery, several randomized trials and meta-analyses have consistently reported that LMWHs are more effective than prophylactic UFH (5000 U twice daily) while maintaining similar bleeding rates [22]. LMWHs have also demonstrated comparable efficacy to higher doses of UFH (7500 U twice daily) but with a lower risk of bleeding [108]. A meta-analysis encompassing trials involving medical and surgical patients revealed that LMWHs reduced the rate of asymptomatic DVT compared to prophylactic UFH, resulting in a 20% relative risk reduction without an increase in major bleeding. These findings were consistent across subgroups of total knee arthroplasty, total hip arthroplasty, and hip fracture surgery [22]. Regarding aspirin, multiple studies investigating its efficacy for thromboprophylaxis after major orthopedic surgery have suggested that it may be less effective than LMWHs. However, aspirin appears to be associated with lower bleeding rates, making it an attractive option [109,110,111,112,113]. The comparison of LMWHs with mechanical methods such as venous foot pump (VFP), intermittent pneumatic compression (IPC), and graduated compression stockings (GCS) has been limited in terms of available research [114,115,116,117,118]. In one randomized trial involving patients undergoing total knee arthroplasty, LMWHs demonstrated a decreased composite endpoint of DVT or death compared to GCS alone (0.9% vs. 3.2%) [114]. In an additional randomized trial involving 274 patients undergoing total hip arthroplasty, there was a trend toward a lower incidence of proximal DVT in the LMWH group compared to patients using VFP alone (9% vs. 13%) [117]. No significant differences in major bleeding complications were observed, although it is noteworthy that more than 20% of patients experienced difficulty tolerating VFP.

**Direct oral anticoagulants**

Several direct oral anticoagulants, such as dabigatran (a thrombin inhibitor) and rivaroxaban, apixaban, and edoxaban (factor Xa inhibitors), have received approval and are gaining popularity as alternatives to LMWHs for VTE prophylaxis in major orthopedic surgery [119,120,121,122,123]. These direct oral anticoagulants are recognized as first-line options for VTE prophylaxis in patients undergoing total hip or knee arthroplasty. However, caution is advised when considering their use in patients who have undergone hip fracture surgery, as there are limited available data for this specific population. It is worth noting that meta-analyses of randomized trials conducted on patients undergoing total knee or hip arthroplasty have yielded evidence indicating that direct oral anticoagulants demonstrate similar efficacy and safety profiles to LMWHs [30,31,32,47,97,124,125,126,127,128,129,130,131,132,133,134,135,136,137,138,139,140,141,142,143,144]. Importantly, reversal agents such as andexanet alfa (for rivaroxaban and apixaban) and praxbind (idarucizumab for dabigatran) are also available to effectively reverse the action of direct oral anticoagulants in the case of serious bleeding.

Direct oral anticoagulants offer a significant advantage in that they are orally bioavailable, unlike LMWHs. Moreover, unlike warfarin, they do not require continuous monitoring and adjustment. Another notable benefit is their suitability for use in patients with a history of HIT. However, it is important to note that direct oral anticoagulants should be avoided in patients with severe renal insufficiency. The recommended dosing for each direct oral anticoagulant used in major orthopedic surgeries is listed in Table 2 [30,31,32,47,97,124,125,126,127,128,129,130,131,132,133,134,135,136,137,138,139,140,141,142,143,144]. The appropriate dosing of direct oral anticoagulants in obese patients remains uncertain. However, according to the guidelines provided by the International Society on Thrombosis and Haemostasis, patients with a BMI greater than 40 kg/m² or a weight exceeding 120 kg may be considered for standard dosing of rivaroxaban and apixaban [145].

In various randomized trials assessing the effectiveness of direct oral anticoagulants, the incidence of symptomatic DVT has generally been comparable to or lower than that observed with LMWHs. While most analyses have indicated no significant difference in bleeding rates, a few studies have reported a slight increase in the risk of bleeding associated with direct oral anticoagulants when compared to LMWHs [30,31,32,47,97,124,125,126,127,128,129,130,131,132,133,134,135,136,137,138,139,140,141,142,143,144]. 

In clinical trials involving patients undergoing total knee and total hip arthroplasty, it has been reported that rivaroxaban demonstrates comparable or superior efficacy compared to LMWHs [30,47,130,131,132,133,134,135]. While some trials indicated a possible increase in bleeding risk, this finding was not consistently observed. Randomized controlled trials have also evaluated the efficacy of dabigatran in patients undergoing total knee and total hip arthroplasty [31,136,137,138,139,140]. A meta-analysis of these trials reported similar efficacy and safety of dabigatran when compared with LMWHs [138]. Specifically, a meta-analysis, evaluating dabigatran and an LMWH, did not find significant differences in rates of symptomatic VTE (RR 0.71; 95% CI 0.23–2.12) or clinically relevant bleeding (RR 1.12; 95% CI 0.94–1.35) [127]. Likewise, randomized trials involving patients undergoing total knee and total hip arthroplasty reported comparable rates of VTE between apixaban and LMWHs without an increased risk of bleeding [32,146,147,148]. Information pertaining to the use of edoxaban for VTE prevention is derived from two randomized trials conducted on Japanese patients undergoing total knee arthroplasty or total hip arthroplasty [141,142]. In a pooled analysis of the two trials, edoxaban demonstrated a lower occurrence of VTE compared to LMWHs (5.1% vs. 10.7%), while exhibiting a comparable safety profile [149]. However, edoxaban has not received regulatory approval for the prevention of VTE.

In a meta-analysis of 22 randomized trials (*N* = 32,159) that compared factor Xa inhibitors (rivaroxaban, apixaban, or edoxaban) with LMWHs for VTE prevention following knee or hip arthroplasty, there was less symptomatic DVT in the direct oral anticoagulant group (4 fewer events/1000). However, that was at the expense of an increased risk of bleeding (2 more bleeding events/1000) [126]. In a subsequent network meta-analysis, patients who received factor Xa inhibitors had a lower incidence of DVT compared with LMWHs (OR 0.45; 95% CI 0.35–0.57) without an apparent increase in the rate of bleeding (OR 1.21; 95% CI 0.79–1.90) [97].

Direct head-to-head clinical trials comparing the various direct oral anticoagulants have not been conducted, thus making it challenging to make direct comparisons between them. However, when a network meta-analysis methodology was employed to indirectly compare these agents, different conclusions were drawn. In a network meta-analysis incorporating data from six randomized trials, which included rivaroxaban (two trials; *N* = 8255), apixaban (one trial; *N* = 5395), dabigatran (two trials; N = 7400), and edoxaban (one trial; *N* = 8240), no significant differences were observed among the various direct oral anticoagulants in terms of the risk of VTE [150]. The risk of major or clinically significant bleeding was lower with apixaban compared with rivaroxaban (RR 0.47; 95% CI 0.36–0.61), dabigatran (RR 0.69; 95% CI 0.51–0.94), and edoxaban (RR 0.54; 95% CI 0.41–0.69). Surprisingly, dabigatran was also associated with a lower bleeding risk compared with rivaroxaban (RR 0.68; 95% CI 0.53–0.87) and edoxaban (RR 0.77; 95% CI 0.60–0.99).

**Aspirin (acetyl salicylic acid)**

Aspirin, also known as acetyl salicylic acid, functions as an inhibitor of cyclooxygenases (COX-1 and COX-2), effectively inhibiting platelet activation and aggregation. This mechanism has been proved highly advantageous in the prevention and management of thrombosis risk. At very low dosages (50–100 mg/day), aspirin demonstrates effective antiplatelet activity. Consequently, it is widely utilized as a first-line drug for thromboprophylaxis. The use of aspirin for thromboprophylaxis in patients undergoing major orthopedic surgery is recommended primarily for lower-risk patients. It is typically employed as an extended-duration prophylactic agent following an initial 5-to-10-day course of anticoagulant prophylaxis [33,151]. Using aspirin as the sole initial agent during the early postoperative course is not recommended. However, in select low-risk patients undergoing surgery for lower-extremity trauma, aspirin may serve as an alternative to LMWHs [113,152]. The preferred dosing of aspirin when used as an extended-duration prophylactic agent following an initial 5-to-10-day course of anticoagulant prophylaxis is 81 mg once daily [33]. When aspirin is used as the sole agent, it appears that 81 mg orally twice daily [152,153] or 160 mg once daily is recommended [154]. Higher doses of 325 mg twice daily do not appear to be more effective than lower doses [155,156]. Previously, higher doses of 500 mg three times a day were used; however, the adverse effects were unacceptable.

Randomized clinical trials have provided evidence of the efficacy of aspirin as a prophylactic agent. Among these trials, the EPCAT II trial stands out as the largest study examining the use of aspirin for extended thromboprophylaxis in patients undergoing major orthopedic surgery. The EPCAT II trial involved 3424 patients who underwent total knee or total hip arthroplasty. These patients initially received five days of thromboprophylaxis with rivaroxaban. Following this initial period, they were randomly assigned to receive either aspirin (81 mg once daily) or continued thromboprophylaxis with rivaroxaban for the subsequent days [33]. Patients who underwent total knee arthroplasty received an additional 9 days of therapy, while those who underwent total hip arthroplasty received an additional 30 days of therapy. It is important to note that the patients included in this trial were carefully selected and represented individuals with a lower risk for VTE. They had no other risk factors for VTE, no other indications for long-term anticoagulation, no history of lower limb or hip fracture in the past three months, and their surgery was elective and unilateral. Furthermore, these patients were able to ambulate within 24 h after surgery. Within this specific population, the transition to aspirin after the initial five days of thromboprophylaxis yielded comparable rates of VTE [33,153].

In patients undergoing major orthopedic surgery, especially hip fracture surgery, aspirin has demonstrated a reduction in the occurrence of symptomatic VTE compared to no thromboprophylaxis. The relative risk reduction associated with aspirin use is approximately 30% [152,154]. However, the effectiveness of aspirin compared to anticoagulant thromboprophylaxis (including UFH, warfarin, LMWHs, and direct oral anticoagulants) is still uncertain, as the majority of trials have reported either inferior or similar efficacy outcomes. [112,157].

Additionally, a randomized crossover trial (CRISTAL) involving 9711 patients compared aspirin (100 mg orally/day) with enoxaparin (40 mg SC/day) for 35 days after hip arthroplasty and 14 days after knee arthroplasty [113]. Enoxaparin demonstrated superiority over aspirin in preventing symptomatic VTE at 90 days, with rates of 1.82% compared to 3.45%. There were no significant differences in the rates of major bleeding (<0.5%) or death. The primary factor driving this outcome was enoxaparin’s effectiveness in preventing distal DVT (1.2% compared to 2.4%), rather than proximal DVT (0.2% each) or PE (0.6% compared to 1.1%). It is important to note that this study had limitations, including early termination due to harm, a 5% loss to follow-up among patients, and the lack of blinding of the treatment allocation for hospitals [113]. Likewise, there was no significant difference in the incidence of VTE among patients who received aspirin alone (2.51% for DVT and 1.5% for PE) for a duration of 21 days compared to those who received LMWH (1.71% for DVT and 1.5% for PE) in another trial involving over 12,000 patients with polytrauma who underwent surgical treatment for fractures. The rates of bleeding (14%) and wound infections (<1%) were also similar between the two groups [152]. Moreover, a meta-analysis of 13 randomized trials involving 6060 patients compared the use of aspirin with various anticoagulants (LMWHs, rivaroxaban, and warfarin) in patients undergoing total knee or total hip arthroplasty. The analysis did not find a significant difference in the rate of VTE (RR 1.12; 95% CI 0.78–1.62) or the rate of major bleeding (RR 1.11; 95% CI 0.47–2.59) [157]. In an older trial, warfarin administered for 21 days was reported to be superior to aspirin in the prevention of venographically detected DVT following hip fracture surgery (20% vs. 41%) [76].

### 2.2. Mechanical Methods

Mechanical prophylaxis devices are successful in decreasing the likelihood of VTE in orthopedic surgeries and are suitable for patients who are unable to undergo pharmacological prophylaxis [3]. Nevertheless, when compared with pharmacological prophylaxis, the effectiveness of mechanical prophylaxis alone is generally lower [78,79,80,81,158]. Hence, it is crucial to incorporate or transition to a pharmacological agent promptly once adequate hemostasis is determined, the risk of bleeding reaches an acceptable level, and/or any bleeding tendencies have been reversed. Mechanical thromboprophylaxis methods encompass IPC, GCS, and VFP. Among these, IPC seems to be the preferred choice as there is more evidence supporting its utilization in orthopedic patients [3].

In general, multiple small trials conducted on patients undergoing procedures such as total knee arthroplasty, hip fracture surgery, or total hip arthroplasty showed that compared to no thromboprophylaxis, the use of IPC resulted in a decrease in the incidence of DVT [159,160,161,162,163]. As an illustration, in a randomized trial involving 310 patients who underwent total hip arthroplasty, the utilization of IPC resulted in a reduction in venographic DVT rates from 49% to 24%, and proximal DVT rates from 27% to 14% [160]. The support for the use of GCS is derived from indirect data obtained from other surgical and medical patients. However, the outcomes associated with GCS use in orthopedic surgeries are varied and linked to an elevated risk of local skin complications. There has been only a single small trial conducted in orthopedic patients, which demonstrated a similar lack of benefit in relation to the use of GCS. However, GCS is commonly employed in conjunction with pharmacological methods for high-risk patients undergoing orthopedic surgery [164]. Multiple small studies have indicated that VFP devices show promise in reducing the occurrence of VTE in patients undergoing significant orthopedic surgery [165,166,167,168,169,170].

Regarding the placement and timing of devices, it is customary to position them on the patient shortly before the commencement of surgery, and they are employed continuously until the patient is discharged from the hospital, begins ambulation, or initiates pharmacological thromboprophylaxis. In cases where it is not feasible to apply a mechanical device to the operated lower extremity, an alternative is to apply it to the opposite extremity to mitigate the risk of DVT. Importantly, routine use of prophylactic inferior vena cava (IVC) filters should be avoided in this context. The risks associated with IVC filter placement are significantly greater compared to the risk of VTE [171,172,173].

## 3. Emerging Drugs: Factor XI(a) Inhibitors

The route of administration, efficacy, safety, drug interactions, and cost of clinically used anticoagulants present both advantages and disadvantages. It is important to note that all existing anticoagulants carry some risk of increased bleeding. Ongoing efforts are underway to develop more effective and safer anticoagulant options. Typically, the evaluation of new anticoagulants begins with dose-finding studies in patients undergoing elective knee arthroplasty. The effectiveness of these agents can be assessed by utilizing venography to determine the incidence of DVT following surgery. This information can then be used to estimate appropriate dosing for other therapeutic uses. One promising target currently being explored is factor XIa [174,175,176].

### 3.1. Factor XIa

Factor XIa is a component of the intrinsic pathway of the clotting cascade. Research has demonstrated that factor XIa plays a significant role in enhancing the generation of thrombin. Individuals who have hereditary factor XI deficiency (also known as hemophilia C) generally exhibit a lower risk of VTE or ischemic stroke compared to those with normal levels of factor XI. In contrast, thrombotic events are significantly increased in the presence of high levels of factor XI. Moreover, individuals with factor XI deficiency rarely experience spontaneous bleeding. Therefore, it has been hypothesized that targeting factor XI/XIa, by blocking the amplification of thrombin generation, might separate thrombosis prevention from normal hemostasis, allowing optimal anticoagulation without causing a significant bleeding risk [177,178,179].

### 3.2. Factor XI(a) Inhibitors in Clinical Trials

Given the promise of factor XI/factor XIa-targeting agents as effective and potentially safer anticoagulants, several molecular entities are in clinical development for VTE prophylaxis in major orthopedic surgeries (Table 3), in addition to other indications [177,178,179].

**Milvexian**

Milvexian is a small-molecule inhibitor of factor XIa that is taken orally, known for its high bioavailability, potency, and selectivity [180,181]. In a randomized trial involving 1242 participants aged 50 years or older who underwent knee arthroplasty, the participants were allocated to receive different doses of milvexian or a once-daily dose of an LMWH (enoxaparin 40 mg) for a duration of 10–14 days after the surgery. Within the study, all participants underwent unilateral venography on the operated leg between 10 and 14 days after the surgery. The findings revealed that individuals assigned to milvexian demonstrated lower rates of VTE. There was a dose-dependent reduction in postoperative VTE rates with once- or twice-daily milvexian. VTE developed in 25% of patients used 25 mg once daily, 21% of patients used 25 mg twice daily, 11% of patients used 50 mg twice daily, 9% of patients used 100 mg twice daily, 7% of patients used 200 mg once daily, and 8% of patients used 200 mg twice daily. In comparison, VTE developed in 21% of patients who received enoxaparin at a dose of 40 mg once daily [182].

The composite incidence of major bleeding, clinically relevant nonmajor bleeding, and minor bleeding was comparable between the two groups, at 4%. Major bleeding was observed in a single participant from the enoxaparin group, while none occurred in the milvexian group. The composite occurrence of major and clinically relevant nonmajor bleeding was 0.8% in the milvexian group and 1.7% in the enoxaparin group. Notably, there was no discernible increase in the rate of clinically relevant bleeding with milvexian across a 16-fold range of doses administered [182].

**Asundexian**

Asundexian is another orally bioavailable, potent, and specific small-molecule inhibitor of factor XIa [183,184,185]. In a randomized trial, a total of 753 individuals diagnosed with atrial fibrillation (with a mean age of 74 years and approximately 33% having chronic kidney disease) were allocated to receive either asundexian (at a dose of 20 or 50 mg once daily) or the factor Xa inhibitor apixaban (at a dose of 5 mg twice daily) for a duration of 12 weeks. The results indicated that participants assigned to asundexian experienced comparable or lower rates of clinical bleeding when compared to those assigned to apixaban [186]. Additionally, the rates of a composite endpoint comprising cardiovascular death, myocardial infarction, stroke, or embolism were found to be 0.8% in the asundexian 20 mg once daily group, 1.5% in the asundexian 50 mg once daily group, and 1.2% in the apixaban 5 mg twice daily group. Notably, no instances of major bleeding were reported. The rates of clinically relevant nonmajor bleeding were 1.2% in the asundexian 20 mg once daily group, 0.4% in the asundexian 50 mg once daily group, and 2.4% in the apixaban 5 mg twice daily group [183,184,185,186]. It appears that asundexian has not been evaluated in orthopedic patients, thus far.

**Osocimab**

Osocimab, also known as BAY 1213790, is a monoclonal antibody that binds in close proximity to the active site of factor XIa, thereby hindering its ability to activate factor IX [187]. Osocimab is classified as an allosteric inhibitor. It is administered intravenously as an infusion lasting over one hour. With a half-life of 30–44 days, osocimab offers the advantage of single-dose administration for surgical prophylaxis. In a study involving 813 adults undergoing elective knee arthroplasty, various aspects including the dose optimization, timing of administration, and efficacy of osocimab were assessed in comparison to enoxaparin and apixaban. Participants were randomly assigned to receive different weight-based doses of osocimab, either preoperatively or postoperatively, or they were assigned to receive enoxaparin (40 mg SC twice daily, initiated either the evening before surgery or 12 to 24 h after surgery) or apixaban (2.5 mg orally twice daily, starting 12 to 24 h after surgery) [188].

Among the participants, the lowest risk of VTE was observed in those who received the highest dose of osocimab (1.8 mg/kg) before the surgery, with a rate of 11.3%. This was followed by the group receiving apixaban, which had a VTE rate of 14.5%. Postoperative administration of various osocimab doses resulted in VTE rates ranging from 15.7% to 23.7%, while the enoxaparin group exhibited a VTE rate of 26.3%. Notably, the majority of VTE events across all groups were asymptomatic, while symptomatic VTE occurred in up to 2% of the participants. The incidence of major bleeding varied depending on the dose and timing of osocimab, ranging from 1% to 4.7%. Apixaban demonstrated a 2% rate of major bleeding, while enoxaparin had a higher rate of 5.9%. Notably, all bleeding events were observed at the surgical site, with no instances of intracranial or other critical site bleeding. Thrombocytopenia was observed in 6.2% of individuals treated with osocimab, 2% of those receiving apixaban, and 5.9% of patients treated with enoxaparin [187,188].

**Abelacimab**

Abelacimab, also known as MAA868, is a monoclonal antibody that specifically targets factor XI as well as its active form, i.e., factor XIa, with high affinity. By binding to factor XI, it effectively immobilizes it in its inactive state, thus preventing its activation by factor XIIa or thrombin. It also neutralizes the catalytic activity of factor XIa. In a trial comprising 412 individuals undergoing knee arthroplasty, the efficacy of three different doses of abelacimab (30 mg, 75 mg, and 150 mg) administered as a single intravenous dose after the surgery was evaluated in comparison to enoxaparin, which was administered subcutaneously once daily at a dose of 40 mg. Some patients also received a single preoperative dose of enoxaparin. The results, as indicated by postoperative venography, demonstrated a lower incidence of VTE with all doses of abelacimab compared to enoxaparin. The postoperative VTE rates were 13%, 5%, 4%, and 22% in the abelacimab groups receiving 30 mg, 75 mg, 150 mg, and enoxaparin 40 mg, respectively. The combined risk of major bleeding and clinically relevant nonmajor bleeding was as low as 2% in the 30 mg abelacimab cohort, 2% in the 75 mg cohort, and 0% in the 150 mg cohort. Similarly, no instances of bleeding were reported among the individuals treated with enoxaparin [189].

**Antisense Oligonucleotides (ASOs)**

ASOs are chemically synthesized oligonucleotides of 12 to 30 bases in length. They are designed to bind specific mRNAs and promote their degradation. Generally, they are administered subcutaneously and are highly bound to plasma proteins with limited glomerular filtration and urinary excretion. Their anticoagulant effect becomes apparent after several weeks, and their long half-life allows reduced frequency of administration. Currently, two ASOs targeting factor XI are under testing, named IONIS-FXIRx [190,191,192] and fesomersen [193,194]. The latter shares with the former the same oligonucleotide sequence but presents with a conjugated N-acetyl galactosamine moiety that increases its hepatic uptake and thus allows monthly instead of weekly administration.

Specifically, IONIS-FXIRx blocks the synthesis of factor XI in the liver, thus decreasing the circulating levels of factor XI [195]. In a randomized, open-label trial, 300 patients undergoing elective knee replacement were assigned to receive IONIS-FXIRx at one of two doses (200 or 300 mg) or enoxaparin (40 mg) once daily. IONIS-FXIRx was started 36 days before surgery and continued up to 3 days after surgery. Both doses were noninferior to enoxaparin, and the highest dose (300 mg) was superior in reducing the incidence of postoperative VTE. The VTE rate, assessed by venography in all patients, was reduced in those receiving ASO 300 mg (4%), compared with ASO 200 mg (27%) or enoxaparin (30%). Bleeding was 3% for ASO 300 mg group, 3% for ASO 200 mg group, and 8% for enoxaparin group [195].

### 3.3. Potential of Factor XIa Inhibitors in Orthopedic Surgeries

A recent meta-analysis of randomized clinical trials comparing the use of factor XI(a) inhibitors versus LMWHs for thromboprophylaxis in patients undergoing major orthopedic surgery demonstrated significant benefits. Factor XI(a) inhibitors were found to be associated with a significant reduction in the incidence of VTE as well as major or clinically relevant nonmajor bleeding events [196].

The findings of the study revealed that the inhibition of factor XI(a) was linked to a substantial reduction, approximately 50%, in both asymptomatic and symptomatic venous VTE events. This reduction is particularly significant considering that the analysis encompassed phase II studies, which typically involve lower dosages of the inhibitors that may not be fully therapeutic. In the sensitivity analysis, which incorporated optimized dosages of factor XI(a) inhibitors, the magnitude of the relative risk reduction for VTE events increased to approximately 75%. In comparison, the incidence of VTE in patients receiving LMWHs was 23.6% in the reported meta-analysis, 27.8% in the Pentasaccharide in Major Knee Surgery Study, and 18.9% in the Regulation of Coagulation in Orthopedic Surgery to Prevent Deep Vein Thrombosis and Pulmonary Embolism (RECORD) 3 study [196]. 

Furthermore, factor XI(a) inhibitors also demonstrated a significant reduction in the risk of major or clinically relevant nonmajor bleeding compared to LMWHs. The magnitude of relative risk reduction was estimated to be around 55%, primarily attributed to a reduction in clinically relevant nonmajor bleeding events. In the sensitivity analysis that compared the relative benefits of more potent dosages of factor XI(a) inhibitors to LMWHs, the decrease in bleeding events was approximately 39%. The incidence of the bleeding events in patients receiving LMWH was 3.2% in this meta-analysis, 2.7% in the RECORD 3 study, and 4.3% in the Apixaban Dose Orally Versus Anticoagulation with Enoxaparin (ADVANCE) trial. Of significant importance, there were no notable differences in the incidence of adverse events and major adverse events between the groups, indicating no evidence of harm associated with the utilization of factor XI(a) inhibitors [196]. Overall, the results of this analysis indicate that factor XI(a) inhibitors reduce the risk of VTE among patients undergoing major orthopedic surgery while also reducing the risk of bleeding events compared to LMWHs.

## 4. Conclusions

Patients who are scheduled for major lower-extremity orthopedic surgery should undergo a comprehensive evaluation to assess their risk for both VTE and bleeding complications. It is important to note that major lower-extremity orthopedic surgery carries a higher risk of symptomatic VTE compared to most other surgical procedures, with an estimated incidence of approximately 4%. This risk is particularly prominent during the first 7–14 days following surgery. Furthermore, the risk of major bleeding is also elevated compared to other surgeries, with an estimated rate of 2–4%. It is essential to consider additional individual risk factors for VTE and bleeding, as they can further contribute to the baseline risk in each patient [3,5,6,7,19,20,21,22].

In the case of patients undergoing major lower-extremity orthopedic surgery who have a low risk of bleeding, it is recommended to implement pharmacological thromboprophylaxis, either with or without IPC devices, rather than not providing any thromboprophylaxis. The selection of the initial agent for thromboprophylaxis is influenced by factors such as the specific type of surgery being performed and the presence of any comorbidities. In the majority of patients undergoing knee or hip arthroplasty, the recommended initial agents for thromboprophylaxis in the early perioperative period are LMWHs such as enoxaparin or dalteparin [92,93,94,95,96,97,98,99,100,101,102,103,104,105,106,107,108], or direct oral anticoagulants such as rivaroxaban or apixaban [119,120,121,122,123]. In contrast, for the majority of patients undergoing hip fracture surgery, an LMWH is the recommended choice for the entire duration of prophylaxis. In cases where patients have severe renal failure, UFH or warfarin may be considered. Additionally, patients with a history of HIT or a current diagnosis of HIT should receive non-heparin alternatives for thromboprophylaxis. When patients have a significant risk of bleeding that makes pharmacological thromboprophylaxis unsuitable, it is recommended to utilize mechanical methods, particularly IPC devices [159,160,161,162,163].

**Figure 1:**
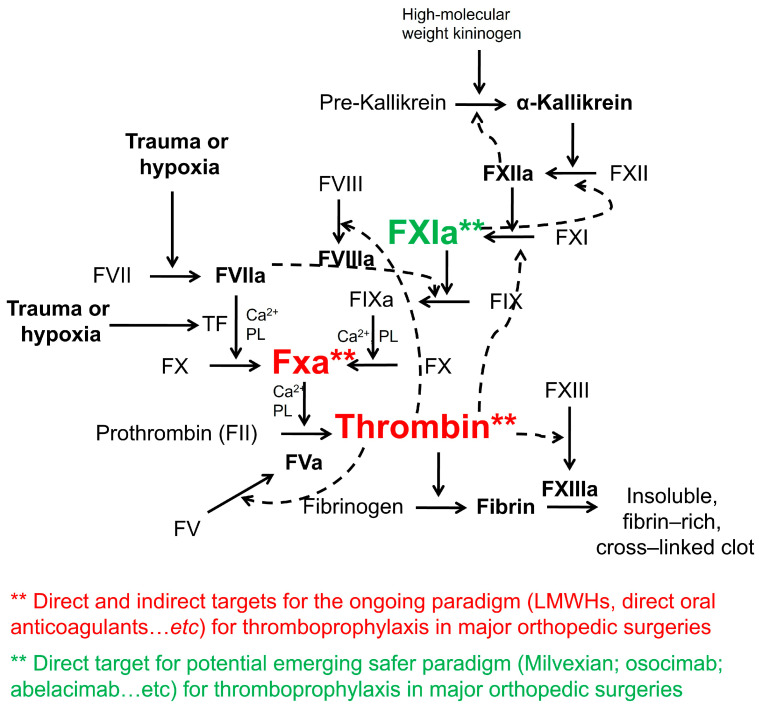
Direct and indirect molecular targets (thrombin and factor Xa) for ongoing paradigm for thromboprophylaxis in major orthopedic surgeries and the direct molecular target (factor XIa) for potential emerging safer paradigm.

Considering emerging therapies, a recent meta-analysis indicated that factor XI(a) inhibitors showed a significant reduction in the incidence of VTE and bleeding events among patients undergoing major orthopedic surgery. This result challenges the ongoing paradigm of anticoagulant therapy in patients undergoing major orthopedic surgeries and suggests that factor XI(a) inhibitors can be a very viable strategy (Figure 1) [196].

## Figures and Tables

**Table 1:** LMWHs for VTE Prophylaxis in Major Orthopedic Surgeries.

LMWH	Use
**Enoxaparin**	In major orthopedic surgeries, 30 mg SC every 12 h or 40 mg once daily started either ≥12 h before or ≥12 h after surgery
**Dalteparin**	5000 U SC daily, starting 12–24 h postoperatively
**Danaparoid**	750 U SC every 12 h, starting 12–24 h postoperatively
**Nadroparin**	38 U/kg SC once daily (maximum: 3800 units) starting either ≥12 h before or ≥12 h after surgery; on postoperative day 4, increase the dose to 57 U/kg once daily (maximum: 5700 units)]
**Tinzaparin**	75 U/kg/d SC, starting 12–24 h postoperatively
**Ardeparin**	In knee surgery, 50 U/kg SC every 12 h, starting 12–24 h postoperatively

**Table 2:** The Recommended Dosing for Oral Direct Anticoagulants in Major Orthopedic Surgeries.

Direct Oral Anticoagulant	Recommended Dosing
**Rivaroxaban**	10 mg once daily, initiated 6 to ≥10 h after surgery
**Apixaban**	2.5 mg twice daily, to be started ≥12 h after surgery
**Dabigatran**	110 mg to be administered one to four hours after surgery, followed by a daily dose of 220 mg thereafter

**Table 3:** Factor XI(a)-Targeting Agents in Clinical Development.

Agent	Type	Administration	Action	Clinical Trials Involving Major Orthopedic Surgeries (Total Knee Arthroplasty)
**Milvexian**	Small molecule	Oral	Inhibitor of factor XIa, by targeting the active site	AXIOMATIC-TKR(Every day or twice-daily oral dosing starting 12–24 h postoperatively for 10–14 days)
**Asundexian**	Small molecule	Oral	Inhibitor of factor XIa, by targeting the active site	It does not appear to have been evaluated in major orthopedic surgeries
**Osocimab**	Monoclonal antibody	Parenteral	Binds to the catalytic domain of factor XIa and inhibits its catalytic activity via allosteric effects	FOXTROT(Single intravenous dose the day after surgery, or just before surgery)
**Abelacimab**	Monoclonal antibody (dual acting)	Parenteral	Binds factor XI, preventing its activation. It also neutralizes factor XIa activity	ANT-005 TKA(Single intravenous dose given postoperatively)
**IONIS-FXIRx**	Antisense oligonucleotide complementary to factor XI mRNA	Parenteral	Reduces the hepatic synthesis of factor XI	FXI-ASO TKA(Nine subcutaneous doses starting 36 days preoperatively. Last dose 3 days postoperatively)

## Data Availability

Provided in the manuscript.
